# 50 years of the Brazilian National Immunization Program and the Immunization Agenda 2030

**DOI:** 10.1590/S2237-96222023000300001.EN

**Published:** 2023-12-04

**Authors:** Jadher Pércio, Eder Gatti Fernandes, Ethel Leonor Maciel, Nísia Verônica Trindade de Lima

**Affiliations:** 1 Ministério da Saúde, Brasília, DF, Brazil

This year, 2023, the Brazilian National Immunization Program (Programa Nacional de Imunizações - PNI) is 50 years old. Established on September 18, 1973 by the Ministry of Health, the PNI became officially recognized in 1975, the year in which Law No. 6,259, dated October 30, 1975 came into force. The law

Provides for the organization of Epidemiological Surveillance actions, with regard to the National Immunization Program, establishes rules relating to compulsory notification of diseases, in addition to providing other measures.^1^ ^),(^ ^2^

Law No. 6,259/1975 was regulated by Decree No. 78,231, dated August 12, 1976,^3^ when the PNI began to coordinate the National Immunization Policy, a public action program fundamental to the formation of the Brazilian National Health System (Sistema Único de Saúde - SUS) in its broadest and most democratic form, as provided for by the 1988 Constitution.^3^

The PNI is responsible for defining the country’s vaccination policy, from the definition of the National Vaccination Schedule to the purchasing of immunobiologicals and their availability in vaccination rooms in the public health service network - more than 38,000 health service facilities -, including the establishment of standards and guidelines on vaccination indications and recommendations throughout the national territory.^4^

In half a century of existence, the PNI has increased the number of immunobiologicals offered to the entire population from 4 to 48 (including vaccines, serums and immunoglobulins). Currently, there are more than 20 vaccines, capable of safely preventing various diseases, available for each stage of life according to the National Vaccination Schedules - children, adolescents, pregnant women, adults, the elderly and special populations (indigenous people and people living with special conditions).^4^

During this period, the commitment of SUS workers, such as the valuable collaboration of experts, researchers, health professionals, managers, partner institutions and the entire Brazilian society, has helped Brazil to achieve high vaccination coverage. Over the years, Brazil achieved control and elimination of several vaccine-preventable diseases, such as measles, poliomyelitis, rubella, congenital rubella syndrome and neonatal tetanus, and the PNI became recognized, nationally and internationally, as a public health success story.^5^

A set of factors has contributed significantly to the progress and relevance of the PNI over these 50 years: vaccination campaigns, such as National Vaccination Day, D-Day against Polio, with a strong communication plan led by the Zé Gotinha character and the support of celebrity immunization ambassadors; strengthening surveillance and diagnosis actions for vaccine-preventable diseases; implementation of pharmacovigilance to monitor vaccine safety; the valuing and expansion of Primary Health Care, as a fundamental SUS strategy for health promotion, disease prevention and control; SUS management decentralization to municipal level; and tripartite administrative management, between the federal administration and the state and municipal health departments.^6^

However, the success of the PNI has been threatened by the recent decline in vaccination coverage, particularly during the COVID-19 pandemic, increasing the risk of reintroduction and spread of vaccine-preventable diseases eliminated in Brazil, such as measles and poliomyelitis.^7^ And this happens precisely when the world is mobilizing to meet the Immunization Agenda 2030 (IA 2030), with the aim of leaving no one behind in terms of being vaccinated.

The PNI offers conditions for Brazil to commit to meeting IA 2030, particularly in an integrated manner with Primary Health Care, through the country’s universal health system, the SUS, and through the national capacity for vaccine production, research and technological innovation. However, the success of IA 2030 in Brazil will depend on political commitment and effective measures, aiming to recover the high vaccination rates achieved over half a century of PNI activities.^8^

At the beginning of 2023 when the current government took office, important changes were implemented in the regulatory structure of the Ministry of Health, including the reformulation and expansion of the Program, whereby its status changed from General Coordination to Department and four new General Coordinations were set up in its current organization chart.^9^ The current administration has established certain priorities to be developed in the coming years, covering strategic actions for (i) recovery of vaccination coverage, (ii) incorporation of new technologies and vaccines, (iii) improvement of information systems, (iv) immunobiological pharmacovigilance, (v) clarification in the face of vaccine hesitancy and combating misinformation, (vi) intensification of vaccine-preventable disease surveillance, (vii) strengthening of the Health Economic-Industrial Complex (Complexo Econômico-Industrial da Saúde - CEIS), (viii) upgrade of the technology park, (ix) modernization of the cold chain and (x) promoting the microplanning strategy.

Microplanning enables the planning and implementation of high-quality immunization activities.^10^ This strategy is an adaptation of the international model adopted by the Pan American Health Organization/World Health Organization (PAHO/WHO) several decades ago, in the Region of the Americas, to ensure high quality vaccination actions, whether in the routine vaccination program or in special strategies such as campaigns, vaccination intensification, tracking, house-to-house vaccination, based on the local reality, with the aim of recovering the high vaccination coverage the PNI historically achieved. Across the country, workshops have been offered for state and municipal health departments to learn about and adopt this strategy in their territories; BRL 151 million have been made available, through a federal ordinance, to support the inclusion and implementation of microplanning in state and municipal health plans.^11^

In its new configuration, the PNI has strengthened ties with various institutions, governmental and non-governmental bodies, and society in general, with the aim of promoting the National Vaccination Movement. This movement, one of the federal government’s priorities in conjunction with state and municipal governments, has the main objective of strengthening the SUS and the country’s vaccination culture.

The PNI’s structure and tradition allow it to commit to IA 2030. Its relevance as an exemplary public health policy worldwide enables the Program to reaffirm its original commitment to reducing the transmission of vaccine-preventable diseases through integrated health surveillance and vaccine pharmacovigilance actions, and health innovation, promotion, prevention and protection actions.

Everyone is invited to celebrate, with just pride, the 50^th^ anniversary of the PNI, which continues to be, and will be even more so, a source of pride for Brazil, hope for the future of our health and expression of the desire of Brazilian men and women to live in a country free from vaccine-preventable diseases.
